# Powering Nutrition Research: Practical Strategies for Sample Size in Multiple Regression

**DOI:** 10.3390/nu17162668

**Published:** 2025-08-18

**Authors:** Jamie A. Seabrook

**Affiliations:** 1 Department of Epidemiology and Biostatistics, Western University, London, ON N6G 2M1, Canada; jseabro2@uwo.ca; Tel.: +1-519-661-2111 (ext. 82234); 2 Brescia School of Food and Nutritional Sciences, Western University, London, ON N6G 2V4, Canada; 3 Department of Paediatrics, Western University, London, ON N6A 5W9, Canada; 4 Children’s Health Research Institute, London, ON N6C 2V5, Canada; 5 Lawson Research Institute, London, ON N6A 4V2, Canada; 6 London Health Sciences Centre Research Institute, London, ON N6A 5W9, Canada

**Keywords:** sample size calculation, multiple regression, nutrition research, statistical power, effect size, beta weights, variance explained, research design, power analysis, multivariable modeling

## Abstract

Robust statistical analysis is essential for advancing evidence-based nutrition research, particularly when investigating the complex relationships between dietary exposure and health outcomes. Multiple regression is a widely used analytical technique in nutrition studies due to its ability to control for confounding variables and assess multiple predictors simultaneously. However, the reliability, validity, and generalizability of findings from regression analyses depend heavily on having an appropriate sample size. Despite its importance, many published nutrition studies do not include formal sample size justifications or power calculations, leading to a high risk of Type II errors and reduced interpretability of results. This methodological review examines three commonly used approaches to sample size determination in multiple regression analysis: the rule of thumb, variance explained (R^2^) method, and beta weights approach. Using a consistent hypothetical example, rather than empirical data, this paper illustrates how sample size recommendations can differ depending on the selected approach, highlighting the advantages, assumptions, and limitations of each. This review is intended as an educational resource to support methodological planning for applied researchers rather than to provide new empirical findings. The aim is to equip nutrition researchers with practical tools to optimize sample size decisions based on their study design, research objectives, and desired power. The rule of thumb offers a simple and conservative starting point, while the R^2^ method ties sample size to anticipated model performance. The beta weights approach allows for more granular planning based on the smallest effect of interest, offering the highest precision but requiring more detailed assumptions. By encouraging more rigorous and transparent sample size planning, this paper contributes to improving the reproducibility and interpretability of quantitative nutrition research.

## 1. Introduction

There are many factors that need to be considered when embarking on a quantitative nutrition research project, including study design, objectives, hypotheses, sampling strategy, operationalization and measurement of independent and dependent variables, sample size, and statistical methods that will be used to analyze and interpret the study’s findings. Multivariate analytical techniques are widely utilized in observational nutrition studies [1,2,3,4,5] due to the necessity of adjusting for participants’ sociodemographic characteristics—such as age, sex, and race/ethnicity—when assessing the strength of an association between an independent variable and dependent variable. This adjustment is crucial to prevent spurious results (i.e., findings that appear to show a relationship between two variables but are actually due to an external factor or random chance) and to ensure accurate interpretation of the exposure-outcome relationships [4]. In a review of statistical reporting and procedures in articles published in the Canadian Journal of Dietetic Practice and Research between 2010 and 2019, Schaafsma et al. [6] found that chi-square tests (*n* = 43), t-tests (*n* = 40), and regression models (*n* = 39) were the three most frequently used statistical tests in quantitative and mixed-method research. While these tests serve different purposes, regression modeling offers several advantages over the others. It provides a more nuanced understanding of the relationships between variables by allowing for the prediction of continuous outcomes and the inclusion of multiple predictors, whereas chi-square tests and t-tests are typically limited to assessing associations between categorical or binary variables and group differences, respectively. This makes regression more flexible for analyzing complex data patterns.

Multiple regression models the relationship between a dependent variable and two or more independent variables (i.e., covariates) by fitting a linear equation to observed data (e.g., estimating coefficients that best describe the relationship). For example, if researchers are interested in assessing the correlation between dietary intake as an independent variable and body mass index (BMI) as a dependent continuous variable, they might assess factors such as caloric intake, protein intake, and fiber intake, while controlling for potential confounding variables such as age, sex, physical activity level, socioeconomic status, race/ethnicity, and sleep duration. This statistical analysis enables researchers to predict a dependent variable using multiple independent variables, control for confounding factors, model complex relationships, and assess how well the predictors explain the variance in the outcome.

Despite the advantages associated with multiple regression (e.g., assessing how multiple factors can affect the outcome, improved accuracy, identification of key factors, and controlling for other influences), the reliability, validity, and interpretability of these models can be undermined by an inadequate sample size. In their review of the literature, Schaafsma et al. [6] found that 25/48 (52.1%) nutrition research studies did not justify their sample size and that 26/33 (78.8%) studies did not include a sample size calculation that required one. This is problematic because inadequate statistical power attenuates the ability to detect significant relationships between variables, limits generalizability to the broader population, and increases the risk of Type II errors (false negatives), which could ultimately be a waste of resources when conducting research [7].

Given the frequency of multiple regression analyses in nutrition research—and the persistent issue of statistical underpowering [8,9]—this review presents three practical approaches for justifying sample size in regression modeling: the rule of thumb, the variance explained (R^2^), and the beta weights approach. I illustrate how each method operates, the assumptions it relies on, and how it can be applied based on a study’s analytic goals and constraints. Rather than a systematic or scoping review of the literature, this methodological review applies these sample size estimation methods to a single, consistent hypothetical nutrition example to demonstrate how recommended sample sizes can differ depending on the chosen approach. A consistent hypothetical example was chosen to isolate and demonstrate the distinct assumptions and implications of each sample size estimation method in a controlled manner, free from variability inherent in empirical datasets or published studies. While this approach enhances clarity and comparability, it also involves trade-offs in terms of generalizability and real-world complexity, which are important to consider when applying these methods to empirical research.

## 2. Materials and Methods

### 2.1. Fundamentals of Sample Size Calculations

In regression analysis, sample size refers to the number of observations or data points (i.e., the individual units of data collected) used to estimate the relationships between variables. For example, if a researcher is investigating how the daily intake of fruits and vegetables affects BMI while controlling for physical activity level, each participant in the study would have an observed value for daily fruit intake, daily vegetable intake, physical activity level, and BMI, which would constitute a single observation.

The sample size required for multiple regression analyses is primarily influenced by four key factors: (i) the number of independent variables (predictors) included in the model, (ii) statistical power, (iii) significance level, and (iv) effect size. In general, as the number of independent variables increases, a larger sample size is required to accurately estimate the regression coefficients and maintain statistical power (i.e., the probability of correctly rejecting the null hypothesis) [10,11,12,13]. While a common target for statistical power in sample size calculations is 80%, an a priori decision to have higher statistical power (e.g., 90%) would necessitate a larger sample size. This is because a higher power means a greater chance of detecting an effect if it exists and reduces the chance of a Type II error. Similar to statistical power, the significance level also influences the sample size. While the threshold for statistical significance is commonly set at 0.05 (5%), a more stringent significance level (e.g., 0.01) would require a larger sample size to achieve the same power. This is because a researcher requires stronger evidence against the null hypothesis to declare a result statistically significant, thereby reducing the probability of a Type I error (i.e., rejecting the null hypothesis when it is true). Lastly, the expected size of the relationship between the predictors and the outcome variable is also very important, such that smaller effect sizes require larger samples to detect statistically significant results, whereas large effect sizes would require smaller samples to obtain a statistically significant finding [10,11,14]. Smaller effect sizes indicate a weaker true relationship between the independent and dependent variables, making it harder to detect, thus necessitating a larger sample size.

There are two commonly held misconceptions regarding sample size considerations in multiple regression analyses. The first misconception is that a larger size is always better. The reality, however, is that excessively large samples can lead to statistically significant results that may not be practically significant [11,12]. The second misconception is that a small sample size does not matter if one obtains a statistically significant result. Although results may be statistically significant, small samples can lead to inflated effect sizes and unreliable estimates, which may not hold in larger, more representative samples [15,16,17,18].

In addition to these core parameters, multiple regression relies on several key assumptions that can influence the accuracy and efficiency of estimation, and as a result, sample size requirements. These assumptions include linearity (the relationship between predictors and the outcome is linear), homoscedasticity (constant variance of residuals across levels of predictors), independence of errors (observations are not autocorrelated), and multicollinearity (predictors are not highly correlated with one another). Violations of these assumptions can reduce statistical power and inflate the standard errors of the estimated coefficients, thereby increasing the sample size required to detect significant effects. For example, multicollinearity can distort the apparent precision of regression estimates by increasing the variance inflation factors (VIFs), which results in wider confidence intervals and reduced statistical significance. Researchers should check these assumptions during study planning and, where possible, adjust their sample size calculations accordingly.

### 2.2. Approaches to Sample Size Calculations for Multiple Regression

Although multiple approaches exist for estimating sample size in multiple regression, three methods are particularly common: the rule of thumb, the variance explained (R^2^), and the beta weights method, with the rule of thumb being the most frequently applied. Each of these approaches is described in detail in the following sections.

## 3. Results

### 3.1. The Rule of Thumb

The rule of thumb recommends including at least 10 participants for every predictor variable in a multiple regression model and is widely cited due to its simplicity and ease of application [6,10]. This rule can be expressed mathematically as

N ≥ 10 × *k*

where:

- N = the total sample size (i.e., the number of participants or observations in the study)
- *k* = the number of predictor variables (also called independent variables or regressors) in the multiple regression model

Returning to the example provided earlier, suppose that a group of researchers were assessing the influence of caloric, protein, and fiber intake on BMI while controlling for age, sex, physical activity level, socioeconomic status, race/ethnicity, and sleep duration. One might assume, based on this example, that there are only three predictor (independent) variables—caloric intake, protein intake, and fiber intake—but this is not the case. Instead, there are three dietary predictors (i.e., caloric intake, protein intake, and fiber intake) and six control variables (i.e., age, sex, physical activity level, socioeconomic status, race/ethnicity, and sleep duration), so the total number of predictors (*k*) is: *k* = 3 (dietary variables) + 6 (control variables) = 9 predictor variables. Since the rule of thumb maintains that at least 10 participants are needed for every predictor variable in the model, and *k* = 9, then:

N ≥ 10 × *k*

N ≥ 10 × 9 = 90

In other words, according to the rule of thumb, a minimum of 90 participants (subjects/cases) is required for this study.

Although widely used, this rule has some important limitations. One limitation is that it cannot ensure that a study is sufficiently powered to detect meaningful effects because the rule is not derived from formal power analysis [19]. More specifically, the rule assumes that effects are of medium size, that predictors are not highly correlated (i.e., low multicollinearity), and that underlying assumptions of the regression model have been met. Furthermore, the rule does not allow researchers to determine how many participants are needed to achieve a particular level of statistical power (e.g., 80%, 90%, or 95%), nor does it account for varying effect sizes or levels of measurement error.

Therefore, while the rule of thumb provides a useful starting point and a conservative minimum estimate of the required sample size, it is best not to be used in isolation. For example, in studies where researchers anticipate small effect sizes, suspect multicollinearity, or aim to achieve a predefined level of statistical power, more sophisticated approaches to sample size estimation are recommended [20,21]. Ultimately, the rule of thumb is best viewed as a practical guideline, rather than a definitive standard.

### 3.2. Variance Explained (R^2^) Method

A second approach to determining sample size in multiple regression involves the anticipated variance in the outcome variable, which in our example is BMI. This variance is typically represented by the coefficient of determination, R^2^, which reflects the proportion of variability in the outcome that is explained by the set of predictor variables.

Continuing with the earlier example, the researchers examine the effects of caloric intake, protein intake, and fiber intake on BMI while controlling for age, sex, physical activity level, socioeconomic status, race/ethnicity, and sleep duration. This resulted in nine predictor variables. If the researchers expect that the nine predictors will collectively explain a modest portion of variability in BMI, such as an R^2^ of 0.13—which Cohen [20] defines as a medium effect size—then power analysis can be used to estimate the required sample size. This approach accounts for several key factors, including the anticipated effect size (R^2^), number of predictors (*k* = 9), significance level, and desired level of statistical power.

Although G*Power was not directly used in this article, sample size estimates based on these parameters were derived to align with standard outputs generated by statistical software such as G*Power 3.1 [21]. For instance, to detect an R^2^ of 0.13 using nine predictors, with a significance level of 5% and statistical power of 80%, G*Power would estimate that approximately 114 participants would be needed. If the goal is to achieve 90% power instead, the required sample size increases to 142 participants.

**Figure 1:**
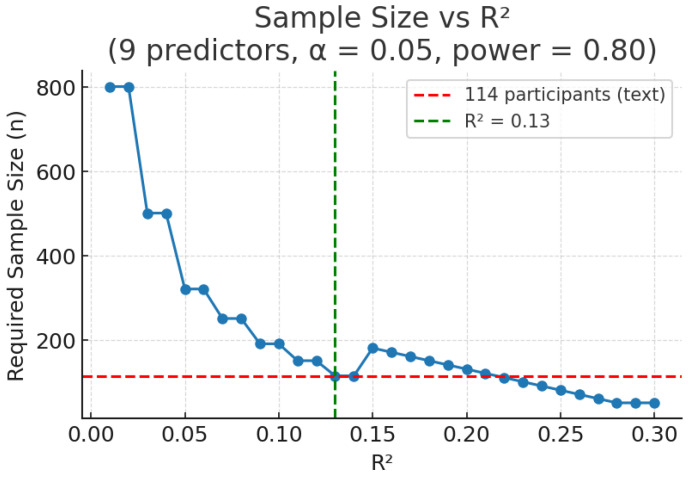
Required sample size by R^2^ for multiple regression (nine predictors, α = 0.05, power = 0.80). Note: Sample sizes were calculated using Cohen’s f^2^ = R^2^/(1 − R^2^) with noncentrality λ = f^2^ × N (G*Power-compatible formulation) [20]. The dashed red line shows the manuscript example value of *n* = 114 at an R^2^ of 0.13. Slightly different conventions for λ (e.g., λ = f^2^ × [N − *k* − 1]) yield marginally higher estimates (e.g., 123 in this example).

These estimates illustrate that when applying the variance explained method to the same research question, the required sample size increases notably compared to the commonly used rule of thumb. While the rule of thumb suggests a minimum of 90 participants for a model with nine predictors (based on 10 participants per predictor), a power analysis assuming an expected R^2^ of 0.13 recommends 114 participants to achieve 80% power and 142 participants to achieve 90% power—an increase of approximately 27% and 58%, respectively. Figure 1 displays a power curve showing how the required sample size decreases as expected R^2^ increases. Expected R^2^ values can be estimated from prior research, pilot data, or meta-analyses. When no prior estimate is available, researchers may consult effect size conventions or conduct sensitivity analysis.

This method has the advantage of tailoring the sample size estimate to the specific design and goals of a study. This is particularly important in confirmatory research settings, where adequate statistical power is essential for detecting meaningful relationships and making valid inferences [20]. Although the rule of thumb can serve as a useful starting point, the variance explained approach provides a more rigorous and context-specific estimate of the required sample sizes.

### 3.3. Beta Weights Approach

A third approach to determining sample size for multiple regression involves considering the magnitude of the beta weights, which represent the unique contribution of each predictor to the outcome variable [12]. Beta weights help researchers understand both the strength and direction of the relationships between the predictor and dependent variables. When estimating sample size, researchers can use the expected size of the beta weights to ensure the study is sufficiently powered to detect meaningful effects.

In multiple regression, beta weights are derived from standardized regression coefficients. They indicate the expected change (in standard deviation units) in the outcome variable for every one standard deviation change in a given predictor, assuming that all other variables in the model are held constant. Larger beta weights suggest a stronger influence of the predictor on the outcome, while smaller beta weights indicate more modest effects.

This approach is particularly relevant when some predictors are expected to have stronger effects than others. Returning to our earlier example, suppose researchers are investigating the influence of caloric intake, protein intake, and fiber intake on BMI while adjusting for age, sex, physical activity level, socioeconomic status, race/ethnicity, and sleep duration. If the researchers hypothesize that dietary factors like caloric intake and protein intake will have relatively large, standardized beta weights—for example, around 0.40 to 0.50—while control variables such as sex or age may have smaller effects, perhaps closer to 0.10 to 0.15, then the sample size calculation can be tailored accordingly. In particular, fewer participants would be needed to detect the larger effects associated with dietary predictors, whereas substantially more participants would be required to reliably detect the smaller effects of the control variables, assuming equal interest in both. Furthermore, when conducting power analyses with varying expected beta weights, researchers can determine the minimum sample size needed to detect each effect with acceptable power (e.g., 80%) and then base their final sample size on the smallest effect of interest. This ensures that the study is sufficiently powered not only to detect large effects but also to detect the smaller, subtler relationships that might be critical for understanding the full model.

**Figure 2:**
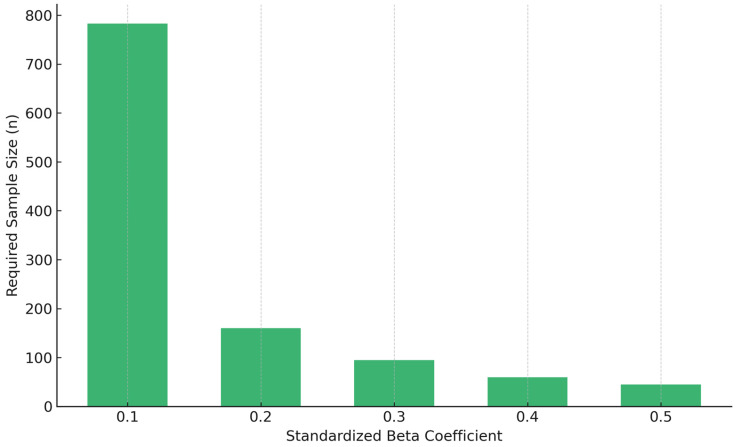
Required sample size by beta coefficient.

To illustrate how expected beta weights influence sample size, a formal power analysis was approximated using the same example, with estimates designed to reflect G*Power outputs. If the smallest effect of interest is a standardized beta of 0.10—which corresponds to a small effect size (f^2^ = 0.01)—a power analysis mirroring G*Power settings (two-tailed test) with nine predictors, an alpha level of 5%, and 80% statistical power would yield a required sample size of approximately 783 participants. If the researchers are only interested in detecting moderate effects, such as a beta of 0.20 (f^2^ = 0.04), the required sample size decreases substantially to about 160 participants. For large effects, such as a beta of 0.50, a sample size of approximately 40 to 70 participants would suffice, depending on the exact model specifications. These estimates highlight the critical importance of anchoring sample size calculations to the smallest effect that researchers aim to detect with confidence. In studies that include both strong and weak predictors—as in this case—basing the sample size on the smallest anticipated beta coefficient provides a more rigorous foundation for ensuring statistical power across all variables in the model. Figure 2 illustrates the relationship between standardized beta coefficients and sample size requirements. Table 1 summarizes required sample sizes across a range of beta values. Researchers can estimate expected beta weights from the literature, theory, or pilot work and should consider sensitivity testing when uncertainty exists.

This method typically involves a more detailed power analysis than the rule-of-thumb or R^2^-based approaches. The researcher must specify an expected standardized beta coefficient for each predictor, along with the total number of predictors in the model, the desired level of statistical power, and the significance level. These parameters can be entered into statistical software, such as G*Power, to estimate the required sample size to detect individual predictor effects, particularly when the goal is to interpret the unique contribution of each variable.

While this approach allows for greater precision and flexibility, it also has limitations. One key challenge is the difficulty in accurately predicting beta weights in advance, particularly when prior research is limited or when high multicollinearity among predictors may cause instability in coefficient estimates. In such situations, the resulting sample size estimates may become less reliable. This approach also assumes that the researcher has a clear and justified hypothesis regarding the expected size of each predictor’s effect, which is not always the case in exploratory analyses.

To help guide sample size decisions when focusing on beta weights, Green [10] proposed a widely cited formula. For studies where the primary interest lies in evaluating the overall variance explained by all predictors—that is, the total R^2^—he recommended a minimum sample size of N ≥ 50 + 8*k*, where *k* is the number of predictor variables. However, when researchers are also interested in examining the statistical significance of individual beta weights, which is often the case in regression analyses, Green suggested an alternative formula: N ≥ 104 + *k*. Applying Green’s guideline to the current example with nine predictors, if the researchers focused only on the overall proportion of variance in BMI explained by the full model, the recommended sample size would be N ≥ 50 + 8(9), which would require at least 122 participants. If, instead, they also wish to evaluate the significance of each individual predictor, the recommended sample size would be N ≥ 104 + 9, which would result in at least 113 participants. While this may seem counterintuitive, it reflects the structure of the formulas: the first is more sensitive to increases in the number of predictors (due to the 8*k* term), while the second adds a fixed constant to allow for hypothesis testing at the level of individual coefficients.

Although Green’s formulas provide widely used approximations for estimating sample size based on medium-sized effects, they are best viewed as practical starting points rather than substitutes for formal power analysis—especially when smaller effects are of interest or when effect sizes vary substantially across predictors. As demonstrated in the power analysis above, detecting small effects, such as a beta of 0.10, may require a sample size far greater than what either of Green’s rules suggests. Ultimately, in studies with a range of expected effect sizes, the most robust approach is to use a formal power analysis based on the smallest meaningful beta weight. This ensures that the study is appropriately powered across all predictors, not just those expected to exert the strongest influence.

In summary, the beta weights approach offers a more precise alternative to rule-of-thumb or R^2^-based methods by aligning sample size with the expected impact of individual predictors. This is especially useful when prior evidence suggests varying effect sizes, ensuring adequate power across all variables. While it requires more detailed assumptions and can be sensitive to multicollinearity, this method provides a rigorous and flexible framework for studies focused on interpreting specific predictor effects.

Table 2 summarizes the required sample sizes derived from each of the three methods described above, based on the consistent hypothetical example used throughout this review.

## 4. Discussion

This review highlights the importance of rigorous and context-sensitive sample size estimation when conducting multiple regression analyses in nutrition research. Although regression techniques are widely employed to control for confounding variables and examine complex exposure-outcome relationships, the reliability and interpretability of these models depend heavily on having an adequate sample size. Our comparison of three common sample size estimation approaches—the rule of thumb, variance in the outcome (R^2^), and expected beta weights—illustrates the trade-offs between simplicity, flexibility, and statistical rigor.

The rule-of-thumb approach, which recommends a minimum of 10 participants per predictor variable, is frequently cited because it is easy to apply [6,10,14]. In our example, which included nine predictors, this method yielded a required sample size of 90 participants. However, this estimate does not account for effect size, statistical power, or multicollinearity, which can significantly influence the validity of regression results [22]. As a result, while the rule of thumb may serve as a useful starting point or conservative minimum, it is insufficient for hypothesis-driven research where precise power estimation is essential. It also assumes medium effect sizes and does not allow researchers to plan based on specific statistical goals. Hanley [23] further emphasized that different research purposes in regression, such as etiological versus predictive modeling, have distinct sample size needs that are not well served by generic rules of thumb.

The variance explained approach offers a more empirically grounded method by linking sample size to the anticipated R^2^ value [20]. Assuming an R^2^ of 0.13 and targeting 80% power resulted in a sample size estimate of 114 participants. Increasing the desired power to 90% raised the estimate to 142 participants. This method enables researchers to tailor sample size planning to the study design and expected effect size. This is particularly useful in confirmatory research. However, it assumes a uniform contribution across predictors and does not provide estimates for the significance of individual coefficients. Riley et al. [24] reinforce the importance of incorporating additional criteria, such as controlling for overfitting and ensuring precision in effect estimates, when determining the minimum sample size for models with continuous outcomes.

The beta weights approach is the most precise of the three because it aligns sample size estimation with the smallest meaningful effect the researcher aims to detect. In our example, detecting a small effect (β = 0.10) required approximately 783 participants, moderate effects (β = 0.20) required 160 participants, and large effects (β = 0.50) required only around 70 participants. This approach is especially valuable when the goal is to interpret the unique contributions of individual predictors rather than assess the overall model fit. However, it requires more detailed assumptions, and estimates may be less stable when multicollinearity is present or when reliable prior information is unavailable [12].

**Figure 3:**
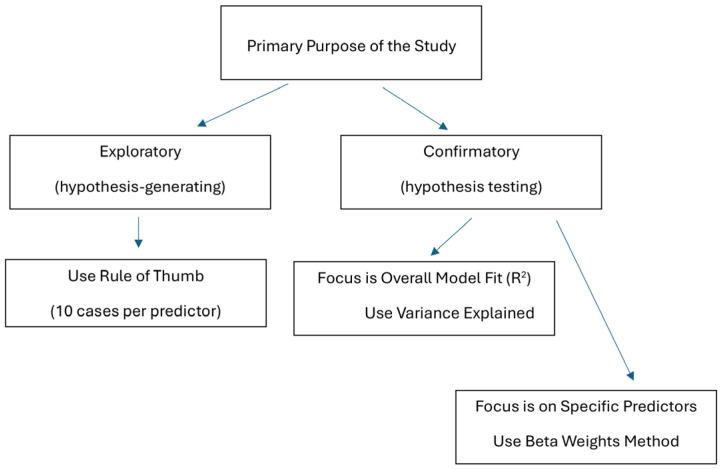
Decision tree for selecting a sample size estimation method in multiple regression.

Figure 3 presents a decision tree for selecting an appropriate sample size estimation method for multiple regression analysis, based on whether the primary purpose of the study is exploratory or confirmatory.

Compared to prior literature, this review provides a practical and accessible comparison of these three commonly used sample size planning methods, grounded in a single example relevant to nutrition science. The goal here is not to promote a single best approach but to clarify how different methods align with distinct research priorities and constraints.

Overall, this review shows that sample size planning should be tailored to the research context. While the rule of thumb may suffice in exploratory studies, more rigorous methods, particularly those based on power analysis, are necessary in confirmatory studies that aim to detect subtle or heterogeneous effects. The choice of approach should be guided by the study objectives, anticipated effect sizes, and tolerance for statistical errors. For example, studies that aim to detect small yet clinically meaningful effects, such as dietary factors influencing BMI, should base their sample size on the smallest expected beta coefficient, rather than general heuristics.

These findings have important implications for nutrition research, where underpowered studies remain common. The lack of formal power calculations in many published studies [6,8] reduces the credibility and generalizability of research findings. Incorporating power analysis into study planning is essential, especially as the field increasingly prioritizes reproducibility and transparent reporting. Researchers should clearly document the rationale for their sample sizes and the assumptions that support their calculations. Although G*Power was not used directly in this review, the sample size estimates were designed to reflect the outputs produced by G*Power using the standard settings. Alternative tools, such as R packages (e.g., pwr, simr) or software like PASS and SAS, can be used for more advanced or customized sample size calculations. Simulation-based methods also offer flexible solutions, particularly for complex models or non-standard assumptions, and can be implemented using platforms such as R and Python. Future work may extend these approaches using simulation-based or Bayesian power analysis methods, which offer flexible alternatives under complex study designs and prior-informed modeling frameworks [25,26]. By broadening the range of accessible tools and encouraging thoughtful planning, these strategies will ultimately improve the interpretability, reproducibility, and impact of nutritional research.

## Figures and Tables

**Table 1:** Sample sizes by beta coefficient.

Beta (Standardized)	Effect Size (f^2^)	Approximate Required Sample Size
0.10	0.011	783
0.20	0.042	160
0.30	0.097	95
0.40	0.267	60
0.50	0.500	45

Note: Estimates were derived to reflect standard outputs from G*Power 3.1, assuming nine predictors, 80% power, and α = 0.05. The required sample size for β = 0.50 varies slightly depending on model specifications; 45 is used here as a representative value.

**Table 2:** Comparison of sample size estimates across methods under varying assumptions.

Method	Assumed Effect Size	Sample Size Estimate
Rule of Thumb	Medium	90
R^2^ Method (R^2^ = 0.02)	Small	395
R^2^ Method (R^2^ = 0.13)	Medium	114
R^2^ Method (R^2^ = 0.26)	Large	63
Beta Weight (β = 0.10)	Small	783
Beta Weight (β = 0.20)	Medium	160
Beta Weight (β = 0.50)	Large	40–70

Note: Sample size estimates were derived to reflect the standard G*Power 3.1 outputs for a model with nine predictors, 80% power, and α = 0.05. R^2^ categories follow Cohen’s [20] guidelines.

## Data Availability

No new data were created or analyzed in this study. Data sharing is not applicable to this article.

## Abbreviations

The following abbreviations are used in this manuscript:

R^2^	Variance explained
BMI	Body mass index
